# Restricted life‐space mobility impacts physical but not mental quality of life in older cancer survivors

**DOI:** 10.1002/cam4.6850

**Published:** 2023-12-22

**Authors:** Richard A. Taylor, Marie Bakitas, Rachel Wells, J. Nicholas Dionne‐Odom, Richard Kennedy, Grant R. Williams, Jennifer Frank, Peng Li

**Affiliations:** ^1^ School of Nursing University of Alabama at Birmingham Birmingham Alabama USA; ^2^ Department of Medicine—Division of Gerontology, Geriatrics, and Palliative Care University of Alabama at Birmingham Birmingham Alabama USA; ^3^ Department of Medicine—Division of Hematology & Oncology University of Alabama at Birmingham Birmingham Alabama USA

**Keywords:** life‐space mobility, older cancer survivors, quality of life

## Abstract

**Background:**

Older cancer survivors often value quality of life (QOL) over survival. Life‐space mobility (LSM), defined as the individual's spatial geographic mobility range, is an important QOL indicator in older adults with chronic illnesses; however, this relationship is unexplored in older cancer survivors.

**Methods:**

We examined the longitudinal associations and causal relationships between LSM and QOL in 153 older cancer survivors (≥65 years) from the University of Alabama at Birmingham (UAB) Study of Aging. LSM was assessed using the UAB Life‐Space Assessment‐Composite score (LSA‐C), and QOL was assessed by the SF‐12 Mental Component Score (MCS12) and Physical Component Score (PCS12) at 0 (study entry), 6, 18, 36, 54, and 72 months. We examined the causal relationship between LSM and QOL using a cross‐lagged panel model (CLPM).

**Results:**

The cohort (*n* = 153) was 76 years old on average and predominantly White (58%), female (58%), and married (55%). Longitudinal analyses found LSM decreased over time (*p* < 0.0001), and this decrease was associated with decreased QOL (PCS12, *p* < 0.0001, MCS12, *p* < 0.0001). In the CLPM causal analysis, lower LSM resulted in worse PCS12 (*p* < 0.001), but not worse MSC12.

**Conclusions:**

Restricted LSM resulted in worse physical QOL over 72 months in a sample of 153 older cancer survivors. Developing and evaluating interventions to preserve greater LSM could be a promising approach to improving QOL.

## Graphical abstract

Life‐space mobility (LSM) represents day‐to‐day mobility. Older cancer survivors have reported that remaining mobile and independent is important to their quality of life (QOL). Restricted LSM has been shown to negatively affect QOL in other diseases in older adults, but it has not been studied in older cancer survivors. Our study in older cancer survivors found that lower LSM was associated with worse physical and mental QOL over time, especially early in cancer survivorship. Furthermore, a causal analysis found that lower LSM resulted in worse physical QOL.

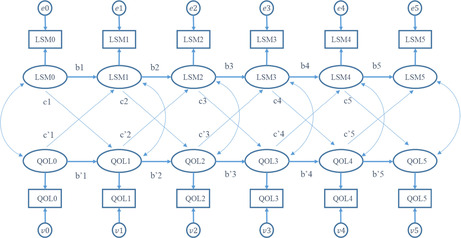

## 1 INTRODUCTION

Sixty‐four percent of US cancer survivors are over 65 years of age, and this number is expected to increase to 71% by 2040.^1^, ^2^, ^3^ The term “cancer survivor” refers to anyone with a history of cancer from the time of diagnosis to death, regardless of treatment or remission status, or whether a cancer‐free status is sustained for many years.^1^ Older cancer survivors often value their QOL above survival, especially regarding their ability to remain fully functional, mobile, independent, and autonomous.^4^, ^5^, ^6^ Cancer and its treatment compound older cancer survivors' functional limitations.^7^, ^8^, ^9^, ^10^ In older adults, decreased function and mobility can result in a high incidence of restricted life‐space mobility (LSM).^11^, ^12^ LSM is a measure of an individual's day‐to‐day spatial range of geographic mobility within their environment, from the home where they sleep to the farthest area they routinely reach by any form (assisted or unassisted) reflecting their social engagement.^11^, ^13^, ^14^ Life‐space mobility depicts the physical and social environment a person inhabits daily, including all forms of mobility (walking, driving, and personal assistance) required to support their desired spatial level of movement and reflects their social engagement.^11^, ^12^, ^13^ LSM requires a continuum of actions to support its purpose and requires executing multiple cognitive and physical mobility‐related tasks such as walking, transferring, driving, or using public transportation.^11^, ^12^, ^13^, ^14^, ^15^, ^16^ Declines or restrictions of mobility limit older cancer survivors' ability to remain independent and autonomous^4^, ^17^, ^18^ and hence it is not surprising that restricted LSM has been associated with poor QOL and health outcomes in older adults with non‐cancer‐related serious illnesses.^19^, ^20^, ^21^, ^22^, ^23^ Older cancer survivors experience more cancer and treatment‐related long‐term sequelae and a range of symptoms (e.g., chronic fatigue, pain, depression, and numbness), have higher rates of comorbidities, poorer overall health, and greater functional limitations, often compounding existing functional limitations, compared to their non‐cancer counterparts or younger adult cancer survivors.^24^, ^25^, ^26^

There is a growing body of literature on the impact of LSM in non‐cancer serious illnesses,^19^, ^20^, ^21^, ^27^ however LSM is understudied in older cancer survivors.^28^, ^29^, ^30^ In a prospective longitudinal mixed methods cohort study, Wong et al.^30^ found that, on average, older non‐small cell lung cancer (NSCLC) survivors had low pretreatment LSM (67.1) when compared to LSM population norms for older adults.^31^ These NSCLC survivors reported a clinically significant decline of 10.1^32^ representing restricted LSM (Life‐Space Assessment [LSA] score < 60) after 1 month of treatment. This decline stabilized but did not rebound to their pretreatment baseline by 6 months.^30^ Qualitative findings from the study indicated fatigue, anxiety, and dependence in Instrumental Activities of Daily Living were associated with the low LSA score;^28^, ^30^ however, that study did not explore the impact of LSM on QOL.

A recent study indicated that assessing LSM using the LSA was helpful in detecting patients with functional decline and subtle functional changes earlier than other standard functional measures in older adult NSCLC survivors.^28^ In this study, 70% of NSCLC survivors had a decline in LSM at 2 months of treatment and at 6 months, only 8% had recovered to baseline, 18% had no recovery, and 37% had additional decline in LSM.^28^ Life‐Space Assessment scores were strongly correlated with QOL scores such that those with lower LSA scores (≥ 10‐point decrease) had lower QOL scores.^28^

There is limited information about the consequences of a declining or restricted LSM in older cancer survivors. The forecast of a vastly increased number of older cancer survivors, the high value they place on their QOL, and the potential to modify LSM support the significance of research into the association of LSM and QOL among older cancer survivors. The aims of this study were to (1) describe the relationship between LSM trajectory and QOL, and (2) explore the longitudinal causal relationship between LSM and QOL. We hypothesized that restricted LSM would be associated with lower QOL over time and that restricted LSM would lead to lower QOL.

## 2 MATERIALS AND METHODS

### 2.1 Study sample

This was a secondary analysis of data derived from the University of Alabama at Birmingham (UAB) Study of Aging, a prospective, longitudinal, observational study of 1000 randomly selected community‐dwelling older adult Medicare beneficiaries in five Alabama counties conducted between 1999 and 2009.^11^, ^13^ The purpose of the parent study was to evaluate the differences in LSM and risk factors for restriction or decline of LSM over time between southern US Black and White participants.^11^, ^13^ Parent study inclusion criteria were as follows: (a) age 65 or older at baseline interview; (b) living independently at baseline in one of selected five counties in the South; (c) Medicare beneficiary; and (d) able to set own study appointments and answer questions by themselves. Parent study participants were selected using stratified random sampling based on county, race, and sex from a list of Centers for Medicare and Medicaid Services (CMS) beneficiaries aged 65 years or older at the time of the first interview (1999 to 2001) residing in three rural and two urban counties in Alabama.^11^, ^33^ The study oversampled for males, AAs, and rural residents.^11^, ^33^ Other inclusion criteria were as follows: (a) living independently and (b) able to set own study appointments and answer questions by themselves. Individuals with mild cognitive impairment/dementia were not specifically excluded.

A total of 3100 persons were mailed a letter including a phone number to call to opt out, 2188 participants were contacted for an in‐home assessment, and 1000 were recruited after refusals and ineligibility were determined.^11^, ^33^ The recruitment goal was 1000 participants, with this goal being set to attain a balanced sample concerning race, gender, and rural/urban county residence.^11^, ^33^ Letters were mailed in sets of 50–100 until the balanced recruitment goal was reached.^11^ Ten days following mailing, up to 10 phone calls were made on different days for 6 weeks to recruit participants.^11^ Data were collected for 102 months. Baseline data were collected for 1000 participants face‐to‐face in their homes. Subsequent data were collected every 6 months over the telephone. There was attrition of participants over the 102 months, with only 381 individuals completing all data collection. The current study included a subset of 153 older cancer survivors (except for any diagnosed only with non‐melanoma skin cancer) before enrollment in the parent study. Due to attrition over time, data from the first 72 months were analyzed. The current study was approved by the UAB (IRB# 300003634) institutional review board.

### 2.2 Measures

All outcomes measures of interest, except demographics, were collected at 0 (study entry), 6, 18, 36, 54, and 72 months.

#### 2.2.1 Life‐space mobility

Life‐space mobility was measured by the total composite score of the UAB Life‐Space Assessment (LSA‐C).^11^, ^13^, ^34^ The LSA‐C measures mobility during the previous month in five different locations or life‐space levels: the home, outside the home, the neighborhood, the town, and beyond. Scoring is based on the level achieved, the frequency, and the need for assistance. The composite score ranges from 0 (bedbound) to 120 (able to travel out of town daily without assistance).^31^ A score less than 60 is considered to indicate restricted LSM.^11^ A change of 5 points in the LSA‐C has been reported as a minimum clinically important difference.^32^ The LSA‐C index has well‐established reliability and validity.^13^, ^34^, ^35^

#### 2.2.2 Quality of life

QOL was measured using the Medical Outcomes Study 12‐Item Short‐Form Health Survey (SF12) questionnaire that is comprised of the Physical Component Summary (PCS12) and Mental Component Summary (MCS12) scores. Scores are standardized to the general U.S. adult population (*M* = 50; SD = 10),^36^ where each component's score ranges from 0 to 100, with higher scores indicating better QOL.^37^ The scales for the PCS summary measure include physical functioning, role‐physical, bodily pain, and general health. The scales for the MCS summary measure include vitality, social functioning, role‐emotional, and mental health.^37^

### 2.3 Demographics and clinical characteristics

Sociodemographic variables included age, gender, race, education, and marital status. Cancer‐related variables included cancer type and cancer history. The cancer variable was self‐reported but considered verified if (1) the person was taking a medication for the condition, (2) the person's primary care doctor noted the condition on a questionnaire sent to them, or (3) the condition was documented in the record for hospitalizations. Cancer stage was not collected. Additional model‐based covariates^38^ included: cognitive function measured by Mini‐Mental State Exam (MMSE) and Clock Drawing Task (CLOX); social support (SS) measured by the Arthritis Impact Measurement Scale 2 (AIMS2) subscale “support from family and friends”; and physical variables measured by the Charlson Comorbidity Index, BMI category, and the short performance battery (SPPB) physical performance scores. The environmental variable included residence (rural/urban) and the area deprivation index (ADI). Financial variables included household income and financial strain (perspective on income). The Duke University Religion Index (DUREL) measured the spiritual variable. Psychological variables were measured by the Geriatric Depression Scale (GDS‐15) scores and Arthritis Impact Measurement Scales 2 (AIMS2) anxiety scores.

### 2.4 Statistical analysis

**FIGURE 1:**
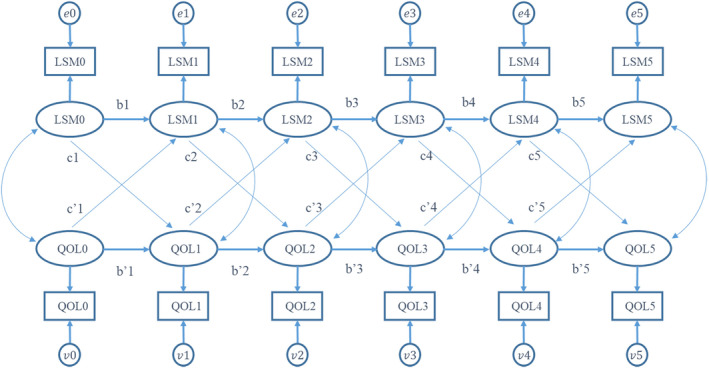
Hypothesized path diagram of a cross‐lagged model for the relationship between LSM and QOL scores over time. Following the SEM notation, circles or ovals indicated the latent variables representing the unobserved true values, while squares indicated the observed variables. LSM0–LSM5 indicated the LSM at 0, 6, 18, 36, 54, and 72 months, respectively. Similarly, QOL0–QOL5 indicated the QOL scores (PCS12 or MCS12) at 0, 6, 18, 36, 54, and 72 months, respectively. The *e*0–*e*5 and *v*0–*v*5 indicated the measurement errors. The single‐headed arrow (→) indicated the assumed direct causal effect, and the double‐headed arrow (↔) indicated correlation (no causal assumption). In above model, b1–b5 and b’1–b’5 were standardized estimates of auto‐regression parameter coefficients, c1–c5 and c’1–c’5 were standardized estimates of cross‐lagged regression parameter coefficients.

All variables were summarized as mean and standard deviation (SD), median and range, or frequency and proportion as appropriate. A general linear mixed regression model (GLMM) was used to examine the longitudinal association between QOL (PCS12 and MCS12) and LSM controlling for the covariates (cognitive function measured by Mini‐Mental State Exam (MMSE) and Clock Drawing Task (CLOX). For significant associations between LSM and QOL scores (MCS12 and PCS12) from GLMMs, the causal relationship between LSM and QOL scores (MCS12 and PCS12) was explored using a cross‐lagged panel model (CLPM). CLPM is a type of structural equation model that estimates and tests the reciprocal (noncausal) and/or directional (causal) relationships between variables over time for panel data.^39^ Briefly, the CLPM assumes that LSM at time *t* is a function of LSM at time t–1 (auto‐regressive effect) and QOL scores at time t–1 (cross‐lagged effect). Similarly, of the QOL scores at time *t* are a function of QOL scores at time t–1 and LSM at time t–1. The CLPM for the relationship between LSM and QOL scores (PCS12 or MCS12) is depicted in Figure 1. In this model, b1–b5 and b’1–b’5 were standardized estimates of auto‐regression parameter coefficients, c1–c5 and c’1–c’5 were standardized estimates of cross‐lagged regression parameter coefficients. The causal predominance can be evaluated by comparing the standardized estimates of the cross‐lagged parameters. For example, to establish LSM ➔ QOL causal relationship we expect that c1–c5 are substantially different from zero and c’1–c’5 are not substantially different from zero. Root mean square error of approximation (RMSEA) and comparative fit index (CFI) were used to evaluate the CLPA model fit. RMSEA <0.05 and CFI >0.9 indicate a good model fit. During the follow‐up, some participants dropped out due to different reasons, for example 136 (89%) finished data collection at 24 months. The missing data of LSM and QOL were handled using the full information maximum likelihood (FIML) method under the assumption of missing at random (MAR). All analyses were conducted using SAS 9.4 (Cary, NC) and R 4.0.2.^40^

## 3 RESULTS

### 3.1 Demographic and clinical characteristics

Participants' (*n* = 153) descriptive statistics are shown in Table 1. Participants had a mean age of 76.05 (6.33). Most participants were White (89 [58%]), female (89 [58%]), married (84 [55%]) or widowed (57 [37%]), and had 7–12 years of education (72 [47%]). The most common cancer types were prostate (*n* = 57 [37.3%]), breast (*n* = 31 [20.3%]), and colon (*n* = 18 [11.8%]) cancer. Most participants were 5 years or more beyond diagnosis (82 [53.3%]). Participants demonstrated mild executive function deficits (CLOX1 mean score 10.4 [3.3]; <11 indicating abnormal), marginal cognitive impairment (CLOX2 mean score 13 [2]; < 13 indicates cognitive impairment; MMSE mean score 25.6 [4.6]; < 24 indicates cognitive impairment). Most participants (82 [53.6%]) had the highest level of social support (16, range 0–16 with a higher score indicating better social support received). The average number of comorbidities was 3.2 (1.7), with most (133 [87.5%]) having two or more. Most were overweight or obese based on BMI (38%, 27%). The average SPPB composite score for physical performance ability was 7.1 (3.3) (range 0–12, higher score indicating better performance). Participants' residential area was approximately evenly distributed between rural and urban. The median area deprivation index (ADI) national ranking was 77 (5–100), which suggested a more disadvantaged neighborhood. Participants were evenly divided about their perception of the adequacy of their income to meet their needs reporting: “just enough to get by on” (45 [29%]), “comfortable, without luxuries” (49 [32%]), and “able to do more or less what they wanted” (49 [32%]). On average, the participants reported being religious or spiritual, as indicated by the DUREL score with a mean of 8 (3.8) and a range of 5–27. Most participants' GDS score was <5 (135 [88%]), and their anxiety score was above 16 (127 [83%]), indicating no anxiety or depression.

**TABLE 1:** Demographic and clinical characteristics (*N* = 153).

Characteristics	Mean (SD) or *n* (%)
Age	76.1 (6.3)
Race	
AA	64 (41.8%)
White	89 (58.2%)
Gender	
Male	64 (41.8%)
Female	89 (58.2%)
Cancer type	
Breast	31(20.3%)
Colon	18 (11.8%)
Lung	8 (5.2%)
Prostate	57 (37.3%)
Other	39 (25.5%)
Cancer history	
1 year or less	22 (14.4%)
1–5 years	49 (32.0%)
5 years or more	82 (53.6%)
Education	
<7 years	26 (17.0%)
7–12 years	72 (47.1%)
≥13 years	55 (35.9%)
Marital status	
Married	84 (54.9%)
Widowed	57 (37.2%)
Separated	3 (2.0%)
Divorced	5 (3.3%)
Never married	4 (2.6%)
MMSE score	
<16	26 (17.0%)
≥16	127 (83.0%)
CLOX1 score	
<11	51 (35.2%)
≥11	94 (64.8%)
CLOX2 score	
<13	36 (24.8%)
≥13	109 (75.2%)
Received social support	
0–9	17 (11.1%)
10–11	11 (7.2%)
12–13	20 (13.1%)
14–15	23 (15.0%)
16	82 (53.6%)
Charlson score	
1	20 (13.1%)
2	36 (23.5%)
≥3	97 (63.4%)
BMI category	
Underweight	9 (6.0%)
Normal	44 (29.1%)
Overweight	58 (38.4%)
Obese	40 (26.5%)
Physical SPPB total score (0–12)	7.1 (3.3)
Residential area	
Rural	73 (47.4%)
Urban	80 (52.3%)
ADI national rank (median, range)	77 (5–100)
Household income based on poverty level for 1999 for two people ≥ 65	
Below poverty	48 (30%)
Above poverty	105 (69%)
Financial strain	
Not enough to make ends meet	10 (6.5%)
Just enough to get by on	45 (29%)
Comfortable but permits no luxuries	49 (32%)
Able to do more or less what wanted	49 (32%)
Spiritual Duke score	8.0 (3.8)
Geriatric depression scale	
0–5 (normal)	135 (88.2%)
>5 (depressed)	18 (11.8%)
Anxiety score	
0–16 (normal)	16 (17.0%)
17–25 (anxiety)	127 (83.0%)

Abbreviations: ADI, Area Deprivation Index; BMI, body mass index; CLOX, executive clock drawing task; SPPB, short performance battery.

### 3.2 Longitudinal association between LSM and QOL

The longitudinal analysis suggested that the LSM significantly decreased over time (*p* < 0.0001). For QOL, the PCS12 scores tended to decline over time, but the decrease was not statistically significant, while the MCS12 scores tended to be stable over time. After controlling for time, demographic, and health‐related covariates, the general linear mixed regression suggested that older cancer survivors with lower LSM had worse QOL, evidenced by lower PCS12 and lower MCS12 scores. Specifically, at any given time during the follow‐up, a five‐point decrease of LSM was associated with a 0.8 unit decrease in PCS12 score (*p* < 0.0001) and a 0.4 unit decrease in MCS12 score (*p* = 0.0001).

### 3.3 Causal relationship between LSM and QOL

**FIGURE 2:**
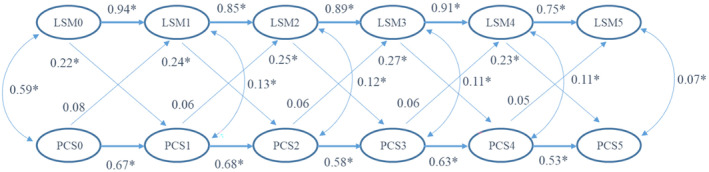
Path diagram with standardized regression coefficient estimates in CLPM to depict the relationship between LSM and quality of life PCS12 scores over time (*p < 0.001).

The CLPM analysis for LSM and PCS12 scores suggested an adequate model fit with RMSEA of 0.06 and CFI of 0.98. The results suggest that all the standardized autoregression coefficient estimates were statistically significant for both LSM (b1–b5) and PCS12 scores (b’1–b’5). The cross‐lagged effects of LSM to PCS12 scores (c1–c5) were statistically significant and substantially different from zero, indicating that the measurement of PCS12 scores at time t could be predicted by the value of LSM observed at time t–1. However, the cross‐lagged effects of PCS12 scores to LSM (c’1–c’5) were neither statistically significant nor substantially different from zero, indicating that the measurement of LSM at time t could not be predicted by the value of PCS12 observed at time t–1. Therefore, the results suggested an LSM‐ > PCS12 causal relationship over time (see Figure 2).

**FIGURE 3:**
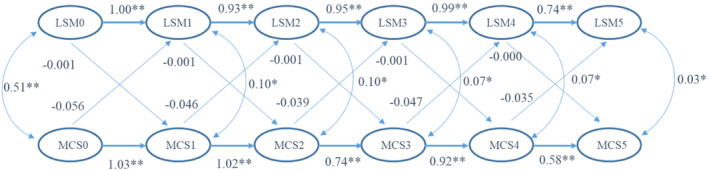
Path diagram with standardized regression coefficient estimates in CLPM to depict the relationship between LSM and quality of life MCS12 scores over time (*p < 0.01; **p < 0.001).

The CLPM analysis for LSM and MCS12 scores suggested an adequate model fit with RMSEA of 0.03 and CFI of 0.99. The results suggest that all the standardized autoregression coefficient estimates were significant for both LSM (b1–b5) and MCS12 scores (b’1–b’5), but none of the standardized cross‐lagged regression coefficient estimates (c1–c5, c’1–c’5) were significant. Because all the standardized cross‐lagged regression coefficient estimates (c1–c5 and c’1–c’5) were close to zero, the causal relationship between LSM and MCS12 scores could not be established (see Figure 3).

The CLPM analysis also suggested that given any time point, the LSM and QOL (both PCS12 and MCS12) were positively correlated (*p* < 0.01) and were consistent with the linear mixed model. More specifically, the strength of the correlation between the LSM and QOL (both PCS12 and MCS12) decreased over time. These results suggested that the early decrease of LSM had more potential effects on QOL.

## 4 DISCUSSION

Our study was one of the first to examine the longitudinal association and the causal relationship between LSM and QOL in older cancer survivors from the UAB Study of Aging. Our results suggested that in older cancer survivors lower LSM was associated with worse QOL over time, and that decreased LSM might explain poorer QOL physical component. Our findings support the importance of early incorporation of LSM assessment into routine oncology care of older adults and the importance of attention to interventional strategies to address factors that may result in LSM declines or restrictions over the cancer survivorship trajectory to avoid poor QOL in this population.

We found an association between LSM and QOL over time; those with lower LSM had worse QOL. Our findings support the limited research on LSM and QOL in older cancer survivors^28^ and the studies of LSM and QOL in other serious noncancer illnesses in older adults.^19^, ^20^, ^21^, ^30^ Restricted LSM in older adults has been associated with poor QOL in all domains.^27^, ^41^ In our older cancer survivors cohort, LSM was affected early in cancer survivorship. The effects of earlier restricted LSM could be explained by acute negative physical/functional impacts of treatment and older cancer survivors' limited physiologic reserve and slower recovery time.^30^ Our findings on LSM are consistent with the extensive literature indicating that older adults, especially those with serious illnesses, experience decreased mobility, increased disability prevalence, and decreased ability to fulfill their daily life activities (especially outside the home), which negatively impacts their QOL, wellbeing, sense of freedom and independence, and social participation, which are all important for healthy aging.^41^, ^42^, ^43^ As noted, a clinically significant decrease of 5 points in the LSM^32^ score was associated with a statistically significant decrease in older adult's physical and mental QOL.

We found a causal relationship over time between older cancer survivors' LSM and the QOL PCS, but not the MCS, indicating that lower LSM levels might cause worse physical related QOL rather than mental. Our findings of no causal relationship between LSM and mental QOL are interesting in that LSM is an indicator of social participation and social functioning (performing normal social activities), a measured concept of mental QOL. Also, previous research in older adults has demonstrated a more significant mediating role of LSM between functional status and mental QOL rather than physical QOL.^36^ Our finding regarding mental QOL may indicate survivors had fewer worries, fears, and distress related to concerns about disease recurrence or new cancer due to their age or the further they got from their cancer diagnosis.^44^, ^45^, ^46^ Also, the mental QOL finding may indicate that, in general, the survivors in our study had less physical, psychosocial, residential area disadvantage, better coping skills, more experience with chronic illness, positive spiritual and religious coping, higher resilience, social connectedness, or good social support and perspective of illness, and protective effects of being married, which could have mitigated the influence of LSM on their mental health.^7^, ^42^, ^47^, ^48^, ^49^, ^50^, ^51^, ^52^, ^53^, ^54^, ^55^, ^56^

To avoid or mitigate the negative consequences related to restricted LSM in older cancer survivors, it is important for healthcare providers to assess LSM routinely and intervene to address modifiable factors that may negatively impact LSM. Interventional strategies to address these modifiable factors impacting the individual's LSM need to be explored. Tracking changes in LSM over time can identify declines early, allowing for assessment and intervention prior to the onset of restricted LSM and its well‐known sequalae. Multiple factors affecting LSM in older adults have been identified and grouped into broad categories (cognitive, social, physical, environmental, financial, sociodemographic, spiritual, psychological, and motility).^18^, ^38^, ^57^ Studies in non‐cancer serious illness have identified factors negatively impacting LSM resulting in poor QOL and health outcomes.^19^, ^20^, ^22^, ^23^, ^58^ There are limited studies of LSM and factors that affect LSM in older cancer survivors, but some of these identified factors include age, new cancer diagnosis, cancer type, physical functioning, perception of income, social support level, number of comorbidities, cognitive functioning, rural residence, incontinence, bereavement, anxiety, systemic treatments, Body Mass Index, unintentional weight loss, fatigue, and muscle weakness.^30^, ^59^, ^60^ Potential interventions, not specific to cancer survivors, have been identified to address issues related to LSM in older community‐dwelling adults based on the categories previously mentioned.^57^ LSM interventions in older adults with serious illnesses are more likely to be beneficial if they are multidimensional and based on the modifiable factors affecting mobility, including motility.^57^, ^61^ In clinical practice, the LSM assessment provides the opportunity to investigate the root causes based on the aforementioned broad categories in order to develop a tailored integrated multidisciplinary approach to mitigation.^61^, ^62^ Some interventions that may be used to increase or maintain LSM in older cancer survivors could include symptom management related to cancer and its treatment toxicities (e.g., pain, fatigue, diarrhea, weight loss, sarcopenia, financial toxicity, depression, and anxiety), physical activities (e.g., exercise interventions focusing on functional capacity and strength, walking, aerobic training, and gait training), psycho‐oncology counseling and support, improving social support networks, diet and nutrition, decreasing environmental barriers, and strengthening/supporting religious/spiritual connectedness.

Our study has several limitations and strengths. First, data were from the Study of Aging collected between 1999 and 2009. Due to changes in treatment and survival rates since the time of data collection, results' generalizability could be limited. Second, we included a heterogeneous sample of older patients with different cancer diagnoses, stages, and treatments. Participants were predominately white, female, and the most common cancer type was prostate cancer which may limit generalizability to other races, genders, and cancer types. Third, the study participants were from one geographic area (Alabama) in the Deep South, which has unique population characteristics, culture, and economics; this may limit its generalizability beyond the Southern U.S. Last, cognitively impaired participants, as indicated by an MMSE score of 16 or less, may not respond accurately to study instruments; therefore, threatening validity. In some individuals, this threat may be mitigated by cognitive resilience.

It is important to note that LSM, as measured by the UAB LSA‐C, and health‐related QOL, as measured by the SF‐12, have some conceptual overlap, but are not measuring the same concept and do not have excessive conceptual overlap. The SF‐12 is one of the most widely used health surveys for measuring overall patient well‐being. The physical health component score (PCS) from SF‐12 measures physical functioning, bodily pain, and general health. The UAB LSA‐C measures the LSM defined as the ability to access different areas extending from the room where the person sleeps to places outside his or her hometown. Two items (“Moderate activities, such as moving a table, pushing a vacuum cleaner, bowling, or playing golf” and “Climbing several flights of stairs”) from the SF‐12 might relate to LSM but are not the same as LSM. Therefore, SF‐12 measures physical functioning more generally, while UAB LSA‐C focuses on a specific physical function—mobility. Previous studies have demonstrated a moderate correlation between SF12 and the LSA^13^ and that physical performance, as measured by the SPPB, accounted for the largest proportion of variance in LSA scores.^63^ These studies demonstrate that the SF12 and the LSA have some overlap but are measuring different underlying concepts, which is what we would expect with measures of QOL (SF12) and mobility (LSA). Theoretically, the PCS from SF‐12 and LSM scores are likely correlated; however, the strength of the correlation can vary greatly depending on different populations, which is the premise of many similar published studies. Our study, enabled by the panel data, focused on the relationship between LSM and QOL (PCS and MCS) in cancer survivors, which has not been explored before in cancer survivors.

Our study was a secondary data analysis in a study that was not originally designed to answer our research question which limited, to some degree, the information specific to LSM. The absence of cancer stage is such a limitation.

The strengths of our study are that it is based on longitudinal data explicitly designed to assess LSM in an older community‐dwelling adult population, which aligns well with the objective of our study. The data included repeated measures of key conceptual factors with long‐term follow‐up. The sample was drawn from the community versus cancer clinics and was racially balanced.

In summary, this study substantially expands the knowledge related to the association of LSM and QOL in older cancer survivors by demonstrating that LSM significantly decreases over time; there was a causal relationship with physical QOL but not mental QOL. Since the number of older cancer survivors is expected to dramatically increase over the next 20 years, increased attention to assessing, promoting, and supporting older cancer survivors' LSM is critically important in maintaining their QOL, especially the physical component. Future prospective research in older cancer survivors needs to focus on identifying these factors and developing and testing specific contextually based interventional strategies unique to older cancer survivors.

## AUTHOR CONTRIBUTIONS

**Richard A. Taylor:** Conceptualization (lead); formal analysis (lead); investigation (lead); methodology (equal); writing – original draft (lead). **Marie A. Bakitas:** Conceptualization (equal); methodology (equal); supervision (equal); writing – review and editing (equal). **Rachel Wells:** Formal analysis (supporting); supervision (supporting); writing – review and editing (equal). **J. Nicholas Dionne‐Odom:** Methodology (supporting); supervision (supporting); writing – review and editing (supporting). **Richard Kennedy:** Conceptualization (supporting); formal analysis (supporting); methodology (supporting); project administration (supporting); resources (equal); supervision (supporting); writing – review and editing (supporting). **Grant R. Williams:** Conceptualization (equal); supervision (supporting); writing – review and editing (equal). **Jennifer Frank:** Project administration (equal); writing – review and editing (equal). **Peng Li:** Conceptualization (equal); formal analysis (equal); methodology (equal); supervision (equal); writing – review and editing (equal).

## EMPLOYMENT INFORMATION

The authors declare they have no employment interests.

## FINANCIAL INTEREST

The authors declare they have no financial interest.

## FUNDING INFORMATION

The American Cancer Society Doctoral Degree Scholarship.

## ETHICS APPROVAL

This study was approved by the University of Alabama at Birmingham Institutional Review Board and was performed in accordance with the ethical standards as laid down in the 1964 Declaration of Helsinki and its later amendments.

## Data Availability

This was a secondary data analysis. Data sharing is not applicable to this article as no new data were created or analyzed in this study.
